# Endothelial function in migraine: a cross-sectional study

**DOI:** 10.1186/1471-2377-10-119

**Published:** 2010-12-01

**Authors:** Floris H Vanmolkot, Jan N de Hoon

**Affiliations:** 1 Department of Internal Medicine, Maastricht University Medical Center, P. Debyelaan 25, 6202 AZ Maastricht, The Netherlands; 2 Center for Clinical Pharmacology, University Hospital Leuven, Leuven, Belgium

## Abstract

**Background:**

Migraine has been associated with cardiovascular disorders. Endothelial dysfunction may be a mechanism underlying this association. The present study tested the hypothesis that endothelium-dependent vasodilation, basal endothelial nitric oxide release and endothelial fibrinolytic capacity are impaired in migraine patients.

**Methods:**

Graded doses of sodium nitroprusside (SNP, 0.2 to 0.8 μg.min^-1^.dL^-1 ^forearm), substance P (0.2 to 0.8 pmol.min^-1^.dL^-1 ^forearm) and N^G^-monomethyl-L-arginine (L-NMMA, 0.1 to 0.4 μmol.min^-1^.dL^-1 ^forearm) were infused into the brachial artery of 16 migraine patients with or without aura during a headache-free interval and 16 age- and sex-matched subjects without a history of migraine. Forearm blood flow (FBF) was measured by strain-gauge venous occlusion plethysmography. Local forearm release of tissue plasminogen activator (t-PA) in response to substance P infusion was assessed using the arteriovenous plasma concentration gradient. Responses to infused drugs were compared between patients and matched controls by analysis of variance.

**Results:**

In both migraine patients and control subjects, SNP and substance P caused a dose-dependent increase, and L-NMMA a dose-dependent decrease in FBF (*P *< 0.001 for all responses). In both groups, substance P caused an increase in t-PA release (*P *< 0.001). FBF responses and t-PA release were comparable between migraine patients and control subjects.

**Conclusions:**

The absence of differences in endothelium-dependent vasodilation, basal endothelial nitric oxide production and stimulated t-PA release between migraine patients and healthy control subjects argues against the presence of endothelial dysfunction in forearm resistance vessels of migraine patients.

## Background

Migraine is a common neurovascular disorder, characterized by recurrent episodes of headache, dysfunction of the autonomic nervous system, and in some patients, an aura involving neurologic symptoms [1]. The pathophysiology of migraine is largely unknown. During the last decade, a neurogenic hypothesis of migraine has largely replaced the classical vascular theory [2]. Nonetheless, migraine has been associated with several cardiovascular disorders including ischemic stroke [3], ischemic heart disease [4], vasospastic disorders [5,6] and genetically determined vasculopathies [7].

The mechanisms underlying the association between migraine and cardiovascular disorders are currently unknown. Migraine has been proposed as an "endotheliopathy" [8]. The vascular endothelium regulates numerous vascular functions [9]. In response to specific stimuli, endothelial cells secrete local vaso-active mediators, including the vasodilator nitric oxide (NO) and vasoconstrictor endothelin-1 (ET-1) and tissue-type plasminogen activator (t-PA), which contributes to the fibrinolytic pathway. Several observations suggest that endothelial function is abnormal is migraine patients, including an increased prevalence of anti-endothelial cell antibodies that may induce endothelial damage [10] and raised plasma levels of ET-1 [11] and von Willebrand factor [12]. Interestingly, ET-1 has been shown to produce cortical spreading depression (CSD) in rats [13]. CSD is considered the biological substrate of the migraine aura and a trigger for migraine attacks [2]. Although evidence from human studies is currently lacking, these observations suggest that an endothelial factor may induce a migraine attack.

The aim of the present study was to test the hypothesis that migraine is associated with endothelial dysfunction. To this end, endothelial function was assessed *in vivo *in the human forearm resistance vasculature by measuring: (1) stimulated endothelial NO release, (2) basal endothelial NO release and (3) stimulated endothelial t-PA release.

## Methods

### Subjects

Migraine patients with or without aura, as defined by the International Headache Society diagnostic criteria [14] and control subjects were recruited from hospital and university staff, university students and the general population through advertisements. Major exclusion criteria were: age < 18 and ≥ 50 years, body mass index (BMI) < 18 and ≥ 30 kg.m^-2^, history of cardiovascular disease, smoking, hypertension (systolic blood pressure > 140 mm Hg or diastolic blood pressure > 90 mm Hg), diabetes, hypercholesterolaemia (total cholesterol > 6.5 mmol/L), pregnancy or lactation and regular use of vasoactive drugs (except hormonal contraceptives). Based on medical history, physical examination, routine laboratory tests and electrocardiography all participants were in good health.

The diagnosis of migraine, initially made by a neurologist or general physician, was confirmed by a validated questionnaire [15]. Patients with a history of migraine of < 1 year, > 15 days of headache per month or using antimigraine drugs > 10 days/month were excluded. Control subjects without a history of migraine were matched for gender, age and hormonal contraceptive use. Control subjects with on average > 1 headache episode per month, a history of moderate or severe headaches or first-degree relatives with a history of migraine were excluded.

The study was approved by the ethical committee of the University Hospital and conducted in accordance with the Declaration of Helsinki. All subjects provided written informed consent prior to participation.

### Experimental conditions

Experiments were performed in the morning after an overnight fast in a quiet, temperature controlled (23 ± 1°C) room. Female subjects were studied on day 5-12 of their menstrual cycle (mid follicular phase) or outside the "pill free" period if taking oral contraceptives. Subjects abstained from any drug (except oral contraceptives) for at least 3 days and from alcohol- and caffeine-containing beverages for at least 24 hours. During the experimental visit, subjects rested supine on a comfortable bed. Migraine patients were headache free for at least 72 hours. If a migraine attack ensued within 24 hours after the visit, measurements were repeated in another headache-free period.

### Intra-arterial drug infusion

A catheter (Angiocath^® ^22G, Beckton Dickinson, Temse, Belgium) was introduced into the brachial artery of the non-dominant arm after local anaesthesia with 1 mL of lidocaine (Xylocaine^®^, 10 mg/mL, Astra Pharmaceuticals, Brussels, Belgium). Drugs or saline (NaCl 0.9%) were infused continuously in a single blind manner using automated infusion pumps (Ivac^® ^P1000, Ivac Medical Systems, Eli Lily, Brussel, Belgium) and pressure-resistant polyethylene infusion tubes (Original-Perfusor^®^, B Braun, Melsungen, Germany). Doses were normalised to forearm volume (measured by water displacement) by maintaining the total rate of intra-arterial infusions constant at 100 μL.min^-1^.dL^-1 ^forearm.

### Drugs

Sodium nitroprusside (SNP, David Bull Laboratories, Warwick, United Kingdom), substance P (Clinalfa, Läufelfingen, Switzerland) and N^G^-monomethyl-L-arginine (L-NMMA, Clinalfa, Läufelfingen, Switzerland) were dissolved in NaCl 0.9% (B Braun, Melsungen, Germany) on the day of the experiment and kept at 4°C. SNP solutions were protected from light during the experiment. Doses were selected based on previous work [16,17].

### Forearm blood flow and blood pressure

Forearm blood flow (FBF) was assessed in both the infused and non-infused arm using strain-gauge venous occlusion plethysmography [18]. Electrically calibrated mercury-in-silastic strain gauges (D.E. Hokanson, Bellevue, USA) were applied at the point of maximal forearm circumference. The hands were occluded from the circulation during measurements through rapid inflation of wrist cuffs to 200 mmHg. Wrist cuffs were inflated at least 60 s before starting plethysmographic recordings in order to allow FBF to stabilize. Upper arm cuffs were intermittently inflated to 40 mmHg for the first 10 s in every 15 s to block temporarily venous return of blood from the forearm and thus obtain multiple plethysmographic tracings (EC6 Plethysmograph, D.E. Hokanson, Bellevue, USA). Analogue voltage output from the plethysmograph was processed by an analogue-to-digital converter (Powerlab^®^/8SP, AD Instruments, Castle Hill, Australia) and appropriate software (Chart^® ^v4.0 for Windows, AD Instruments, Castle Hill, Australia) and recorded onto a computer (Dell Optiplex^® ^G1, Dell Computer Corporation, Limerick, Ireland). Calibration was achieved using the internal standard of the plethysmograph.

**Figure 1:**
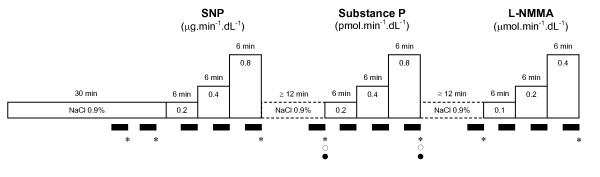
**Study protocol**. SNP, sodium nitroprusside; L-NMMA, N^G^-monomethyl-L-arginine; FBF, forearm blood flow.

Systolic and diastolic blood pressure and heart rate were measured in the non-infused arm using a validated semi-automated oscillometric device (OMRON 705IT, OMRON Healthcare, Hoofddorp, The Netherlands). Measurements were performed immediately after the preceding FBF recording (Figure 1).

### Blood sampling and assay

Arterial and venous blood samples were obtained at the end of substance P infusions and the preceding saline infusion (Figure 1). For venous blood sampling, a catheter (20 or 22 G, Insyte-W, Beckton Dickinson, Temse, Belgium) was inserted into a superficial antecubital vein of the infused arm. Arterial blood was sampled through the arterial catheter. Blood (4.5 mL) was collected into ice-cooled glass tubes containing 0.5 mL 0.105 M sodium citrate (BD Vacutainer^®^, Beckton Dickinson, Plymouth, UK) and kept on ice before being centrifuged at 2500 g for 15 min at 4°C within 1 hour of sampling. Plasma was transferred to plastic tubes (CryoTube^®^, Nunc A/S, Roskilde, Denmark) and stored at -80°C until analysis. Plasma t-PA concentration was determined using an enzyme-linked immunosorbent assay as previously described [19].

### Study protocol (Figure 1)

After arterial cannulation, saline was infused for 30 min to allow for equilibration. At the end of this infusion, two FBF measurements (3 min each), were performed. The mean of these was used as baseline FBF. Three incremental doses of each drug (SNP at 0.2, 0.4 and 0.8 μg.min^-1^.dL^-1 ^forearm; Substance P at 0.2, 0.4 and 0.8 pmol.min^-1^.dL^-1 ^forearm; and L-NMMA at 0.1, 0.2 and 0.4 μmol.min^-1^.dL^-1 ^forearm) were infused for 6 min at each dose, separated by at least 12 min of saline infusion between the different drugs (wash-out). FBF was measured during the final 3 min of each drug infusion or wash-out infusion period. The order of drug infusions was the same for all subjects.

### Data analysis and statistics

FBF was determined from the slope of the initial part of each plethysmographic tracing. Plethysmographic data were extracted from Chart data files and FBF responses were calculated for individual plethysmographic tracings using a template spreadsheet (Microsoft Excel 2000 v9.0, Microsoft Corporation, USA). Tracings unsuitable for analysis due to motion artefacts were manually rejected. All tracings were analysed by the same analyst who was unaware of the subject's diagnosis. The mean of the final 5 FBF tracings from each recording period was used for analysis. The FBF measurement at the end of the saline infusion preceding each drug infusion was used as baseline measurement for that particular drug infusion. FBF responses were expressed as absolute FBF in ml.min^-1^.dL^-1 ^forearm for SNP and Substance P and as percentage change from baseline in the FBF-ratio between infused and non-infused arm for L-NMMA, as discussed previously [20].

As an estimate of local t-PA release, the product of the arteriovenous plasma concentration gradient and forearm plasma flow was calculated: release = (C_v_-C_a_) × FBF × [(101-hematocrit)/100], where C_v _and C_a _are the venous and arterial plasma concentrations, respectively [21].

Comparison of baseline characteristics between groups was made by *t*-test for independent samples. FBF responses, blood pressure and heart rate were analysed by repeated measures analysis of variance (ANOVA), with dose as within subject variable. Comparisons between groups were made by repeated measures ANOVA, with dose as within subject variable and group (migraine or control) as between subject variable. As a summary measure, the mean difference in FBF response between groups, expressed as change in FBF from baseline to the highest infused dose, was calculated together with the 95% confidence interval (CI). Based on power calculations using data from previous work [22], the population size of the present study gives 80% power at a significance level of 0.05 for detecting a 29% difference in FBF increase between groups in response to the highest dose of Substance P.

All statistical analyses were performed using commercially available software (NCSS 2000, Number Cruncher Statistical Systems, Kaysville, USA). *P *< 0.05 was considered statistically significant. Data are presented as mean ± SEM in figures and mean ± SD in tables and text.

## Results

No differences in baseline characteristics were observed between groups (Table 1). Migraine patients had been suffering from migraine for 5.6 ± 3.3 years. Migraine with aura was diagnosed in 9 (56%) and migraine without aura in 7 (44%) patients. Migraine attacks occurred with a frequency of 1.2 ± 0.9 per month and lasted 20 ± 11 hours. As acute anti-migraine drugs, analgesics (paracetamol or non-steroidal anti-inflammatory drugs) were used by 75%, triptans by 13% and metoclopramide by 6% of patients.

**Table 1:** Baseline subject characteristics

Characteristic	Control subjects (n = 16)	Migraine patients (n = 16)	*P*
Age, years	24.0 ± 3.0	24.5 ± 4.0	0.68
Female, n (%)	12 (75)	12 (75)	1.0
Current hormonal contraceptive use, n (% of women)	10 (83)	10 (83)	1.0
Body mass index, kg/m^2^	22.6 ± 2.7	22.7 ± 2.3	0.92
Systolic blood pressure, mm Hg	119 ± 8	117 ± 9	0.47
Diastolic blood pressure, mm Hg	70 ± 5	70 ± 7	0.93
Heart rate, bpm	61 ± 10	64 ± 9	0.29
Total cholesterol, mmol/L	4.0 ± 0.8	4.1 ± 0.5	0.72
HDL cholesterol, mmol/L	1.8 ± 0.4	1.7 ± 0.4	0.80
LDL cholesterol, mmol/L	1.9 ± 0.6	1.9 ± 0.6	0.78
Triglycerides, mmol/L	0.8 ± 0.2	0.9 ± 0.4	0.27
Glucose, mmol/L	4.4 ± 0.2	4.5 ± 0.3	0.81
Forearm volume, mL	919 ± 146	855 ± 154	0.24

Blood pressure, heart rate and FBF in the non-infused arm did not change during the infusions (data on file; Table 2).

**Table 2:** Forearm blood flow

	**Control subjects (n = 16)**				**Migraine patients (n = 16)**			
	
Sodium nitroprusside dose, μg.min^-1^.dL^-1 ^forearm	0	0.2	0.4	0.8	0	0.2	0.4	0.8
non-infused arm	2.0 ± 0.9	1.9 ± 0.7	1.8 ± 0.8	1.8 ± 0.8	2.0 ± 0.5	2.0 ± 0.6	2.1 ± 0.8	1.9 ± 0.6
infused arm	2.0 ± 0.5	8.6 ± 3.5	10.9 ± 4.2	14.5 ± 5.9*	2.1 ± 0.5	8.7 ± 2.9	12.1 ± 4.6	16.5 ± 6.2*
Substance P dose, pmol.min^-1^.dL^-1 ^forearm	0	0.2	0.4	0.8	0	0.2	0.4	0.8
non-infused arm	1.8 ± 0.8	1.8 ± 0.7	1.8 ± 0.7	1.9 ± 0.8	2.0 ± 0.6	1.9 ± 0.6	2.1 ± 0.6	2.0 ± 0.6
infused arm	2.2 ± 0.7	5.5 ± 2.2	6.9 ± 2.5	8.7 ± 3.8*	2.5 ± 0.7	5.1 ± 1.2	6.5 ± 1.8	8.4 ± 2.5*
L-NMMA dose, μmol.min^-1^.dL^-1 ^forearm	0	0.1	0.2	0.4	0	0.1	0.2	0.4
non-infused arm	1.7 ± 0.5	1.8 ± 0.7	1.9 ± 0.9	1.9 ± 0.8	2.1 ± 0.5	1.9 ± 0.6	2.1 ± 0.6	2.4 ± 0.8
infused arm	2.0 ± 0.5	1.7 ± 0.4	1.6 ± 0.4	1.4 ± 0.4*	2.5 ± 0.6	2.0 ± 0.5	1.9 ± 0.6	1.9 ± 0.6*

Data are expressed as absolute blood flows (ml.min^-1^.dL^-1 ^forearm); *P < 0.001 (ANOVA)

**Figure 2:**
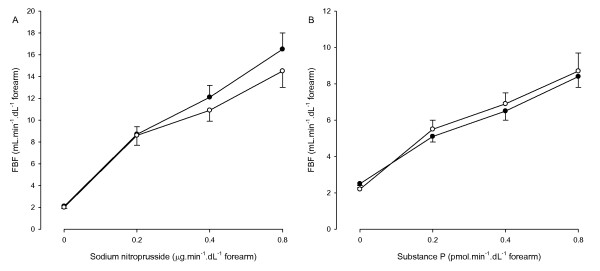
**Forearm blood flow (FBF) response to sodium nitroprusside (A) and substance P (B) in migraine patients (closed circles) and control subjects (open circles)**. *P *< 0.001 for all responses.

SNP and Substance P increased FBF in a dose-dependent fashion (Table 2, Figure 2). The FBF responses to SNP and Substance P were comparable between migraine patients and controls (*P *= 0.44 and *P *= 0.80, respectively). The mean differences in FBF response to SNP and Substance P were 1,8 ml.min^-1^.dL^-1 ^forearm (95% CI, -2.5 to 6.1) and -0,6 ml.min^-1^.dL^-1 ^forearm (95% CI -2.8 to 1.6), respectively.

**Figure 3:**
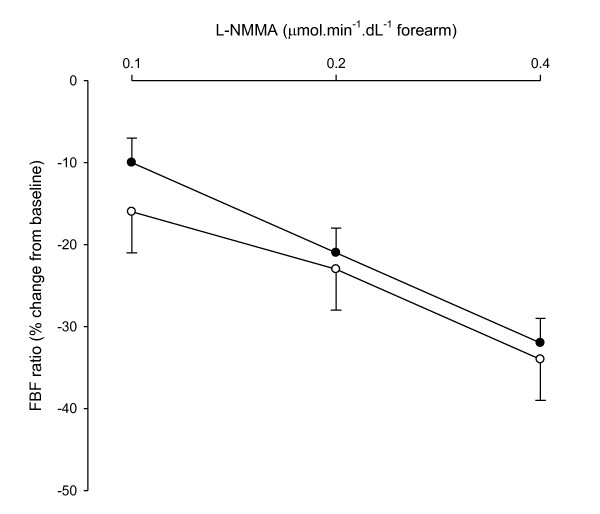
**Forearm blood flow (FBF) response to N^G^-monomethyl-L-arginine (L-NMMA) in migraine patients (closed circles) and control subjects (open circles)**. *P *< 0.001 for all responses.

L-NMMA decreased FBF in a dose-dependent fashion (Table 2, Figure 3). The FBF response to L-NMMA was comparable between migraine patients and controls (*P *= 0.50). The mean difference in FBF response to L-NMMA was 2% (95% CI, -10 to 13).

Venous plasma t-PA levels did not differ between migraine patients and controls (7,4 ± 11 ng.mL^-1 ^and 6,5 ± 5.4 ng.mL^-1^, respectively, *P *= 0.86). The basal and stimulated t-PA release were comparable (*P *= 0.69 and *P *= 0.87, respectively) between migraine patients (-0.6 ± 1.1 ng.dL^-1^.min^-1 ^and 7.4 ± 5.5 ng.dL^-1^.min^-1^) and controls (-0.6 ± 2.1 ng.dL^-1^.min^-1 ^and 7.1 ± 5.5 ng.dL^-1^.min^-1^).

Subgroup analyses did not reveal differences between migraine patients with aura and without aura (data not shown).

## Discussion

In the present study, we compared FBF responses to SNP, Substance P and L-NMMA between migraine patients with or without aura and matched individuals without migraine. In addition, we compared the acute t-PA release in response to substance P in both groups. Our findings indicate that several markers of endothelial function, including the stimulated and basal endothelial NO release and stimulated endothelial t-PA release, are not altered in migraine patients in between migraine attacks.

Endogenous NO is generated by the conversion of the amino acid L-arginine to citrulline by three different nitric oxide synthases (NOS). Neuronal and endothelial NOS are constitutively expressed and produce relatively small quantities of NO in comparison with the inducible form of the synthase. Endothelial-derived NO is a key molecule in vasomotor function. To our knowledge, this is the first study that assessed the basal endothelial release of NO in migraine patients, using a non-selective antagonist of nitric oxide synthase (L-NMMA). The response to L-NMMA did not differ between patients with migraine and control subjects, indicating that the basal endothelial release of NO from peripheral resistance vessels is unaltered in migraine patients, at least outside the migraine attack period. This does not exclude that basal NO release from other vascular beds is altered in the same patients, or that it is impaired preceding, during or immediately after a migraine attack. Previous studies measuring metabolites of NO in platelets or plasma during a headache free interval suggested a basal hyperactivity of the L-arginine-NO pathway [23-25]. Our findings suggest that, if basal NO production is indeed increased in migraine patients, other NO synthases than endothelial NOS in extracranial vessels are implicated.

NO induces vasodilation through a direct action on the vascular smooth muscle cell. A recent study in patients with migraine without aura, showed an impaired FBF response to the NO donor SNP, leading the authors to the hypothesis that vascular smooth muscle cell function is impaired in migraine without aura [26]. In contrast to these findings, we observed a comparable vasodilator response to SNP between patients with migraine and controls, which is in agreement with a previous, smaller study by our group [27]. A small study in patients with migraine without aura also showed a normal response to SNP of the cutaneous microcirculation measured by laser Doppler flowmetry [28]. Adding more complexity are findings from previous work in patients with migraine without aura showing an increased vasodilator response to another NO donor, nitroglycerin, of the brachial artery [29] or intracranial blood vessels [30], suggesting an arterial hypersensitivity to NO. Thus, existing data are clearly inconsistent, which may be explained by differences in patient characteristics (longstanding migraine or migraine of recent onset; migraine with or without aura) or differences in the vascular beds studied (micro- or macrovascular beds, conduit or resistance arteries; intracranial or extracranial arteries).

Several studies have evaluated endothelial function in patients with migraine. The present study confirms the findings of a previous, smaller study by our group (n = 10, 1 patient with aura) that showed no difference in FBF response to serotonin, which releases NO through endothelial receptors [27]. A recent study in patients with migraine without aura (n = 12) assessed endothelial-dependent vasodilation by measuring FBF responses to acetylcholine [26]. Compared to healthy controls, FBF response to acetylcholine was impaired, but as FBF response to SNP, i.e endothelial-independent vasodilation, was impaired while acetylcholine-induced release of NO was intact, the authors hypothesized that vascular smooth muscle cell, but not endothelial cell function is impaired in migraine without aura. A small study in patients diagnosed with migraine without aura (n = 9) showed a normal dermal blood flow response to acetylcholine measured by laser Doppler flowmetry [28]. Two small studies (n ≤ 10) did not observe a difference in flow-mediated dilation (FMD), a measure of endothelium-dependent vasodilation, between patients and controls [27,31]. However, these studies were likely underpowered, considering the large variability of FMD measurements [32]. Recently, a larger study (n = 45) and a smaller follow-up study (n = 24) observed a decreased brachial FMD in patients without aura [29,33]. An impaired FMD was also shown by our group in a group of patients with migraine with or without aura of recent onset (n = 50) [34]. In contrast, a study in patients with migraine with and without aura (n = 50) did not find a difference in FMD between patients with migraine and controls and between patients with migraine with aura (n = 25) and without aura (n = 25) [35]. Taken together, results are clearly inconsistent. The discrepancies between studies may be related to heterogeneity in study population, differences in vascular beds studied and differences in applied endothelial stimulus.

Previous work in patients with migraine suggested abnormalities of haemostasis, including abnormal platelet function, antiphospholipid antibodies and congenital thrombophilia, which may cause increased thrombotic risk in these patients [36]. The endogenous fibrinolytic system and, in particular, the acute release of t-PA from the endothelium protects the circulation from intravascular thrombosis. We assessed the acute fibrinolytic capacity of the endothelium by stimulating the endothelial release of t-PA using intra-arterial infusion of substance P [21]. No difference was observed in the forearm t-PA release between migraine patients and control subjects, suggesting that endothelial fibrinolytic capacity is normal in migraine patients during a headache-free interval. A larger cross-sectional study observed an increased plasma t-PA concentration in young women with (n = 61) and without (n = 64) aura compared to control subjects [37].

The present study has several limitations. First, because of the invasive nature of the forearm model, a relatively small number of subjects was included. In addition, the FBF response to Substance P in the present study was 2 times lower than expected from previous work [22] on which power calculations were based. Therefore, it cannot be excluded that the present study was insufficiently powered to detect small, but clinically relevant differences between migraine patients and control subjects, in particular between migraine subgroups. Interestingly, recent prospective studies suggest that patients with migraine with aura, but not without aura, are at increased risk for cardiovascular events [4,38]. Therefore, additional studies in migraine subpopulations with sufficient power are needed. Second, although the forearm model serves as a convenient model for studying endothelial function, measurement of coronary and cerebrovascular endothelial function is likely to be of greatest clinical relevance, as the forearm vascular bed is less susceptible to atherosclerosis and subsequent thrombosis. Nevertheless, consistent findings between the peripheral and coronary circulation support the notion that the forearm model is a reasonable surrogate [39]. Studies comparing endothelial function in cerebral and peripheral vascular beds in the same patients are lacking. Third, endothelial fibrinolytic capacity depends on the balance between endothelial release of t-PA and plasminogen activator inhibitor type 1 (PAI-1) [21]. As no measurements of PAI-1 or t-PA activity were performed in our study, we cannot completely rule out an impaired endothelial fibrinolytic activity in patients with migraine. Finally, regarding the hypothesis of an endothelial trigger for a migraine attack, we cannot exclude the existence of a local endothelial dysfunction in the cranial circulation.

## Conclusions

In conclusion, the absence of differences in endothelium-dependent vasodilation, basal endothelial nitric oxide production and stimulated t-PA release between migraine patients and healthy control subjects argues against the presence of endothelial dysfunction in forearm resistance vessels of migraine patients.

## List of Abbreviations

CSD: cortical spreading depression; ET-1: endothelin-1; FBF: forearm blood flow; FMD: flow-mediated dilation; L-NMMA: N^G^-monomethyl-L-arginine; NO: nitric oxide; NOS: nitric oxide synthase; SBP: systolic blood pressure; SNP: sodium nitroprusside; t-PA: tissue plasminogen activator

## Competing interests

The authors declare that they have no competing interests.

## Authors' contributions

FV participated in the design of the study, conducted all experiments, performed the statistical analyses and drafted the manuscript. JdH conceived of the study, participated in its design and helped to draft the manuscript. Both authors read and approved the final manuscript.

## Pre-publication history

The pre-publication history for this paper can be accessed here:

http://www.biomedcentral.com/1471-2377/10/119/prepub
